# Exploring the Molecular Basis for Binding of Inhibitors by Threonyl-tRNA Synthetase from *Brucella abortus*: A Virtual Screening Study

**DOI:** 10.3390/ijms17071078

**Published:** 2016-07-19

**Authors:** Ming Li, Fang Wen, Shengguo Zhao, Pengpeng Wang, Songli Li, Yangdong Zhang, Nan Zheng, Jiaqi Wang

**Affiliations:** 1 Ministry of Agriculture Laboratory of Quality & Safety Risk Assessment for Dairy Products (Beijing), Institute of Animal Science, Chinese Academy of Agricultural Sciences, Beijing 100193, China; liming01@caas.cn (M.L.); wenfang@caas.cn (F.W.); zhaoshengguo1984@163.com (S.Z.); woaisanzang@126.com (P.W.); lisongli@caas.cn (S.L.); zhangyangdong@caas.cn (Y.Z.); wangjiaqi@caas.cn (J.W.); 2 Ministry of Agriculture—Milk and Dairy Product Inspection Center (Beijing), Beijing 100193, China; 3 State Key Laboratory of Animal Nutrition, Institute of Animal Science, Chinese Academy of Agricultural Sciences, Beijing 100193, China

**Keywords:** bovine brucellosis, homology modeling, molecular dynamics, virtual screening, docking

## Abstract

Targeting threonyl-tRNA synthetase (ThrRS) of *Brucella abortus* is a promising approach to developing small-molecule drugs against bovine brucellosis. Using the BLASTp algorithm, we identified ThrRS from *Escherichia coli* (EThrRS, PDB ID 1QF6), which is 51% identical to ThrRS from *Brucella abortus* (BaThrRS) at the amino acid sequence level. EThrRS was used as the template to construct a BaThrRS homology model which was optimized using molecular dynamics simulations. To determine the residues important for substrate ATP binding, we identified the ATP-binding regions of BaThrRS, docked ATP to the protein, and identified the residues whose side chains surrounded bound ATP. We then used the binding site of ATP to virtually screen for BaThrRS inhibitors and got seven leads. We further characterized the BaThrRS-binding site of the compound with the highest predicted inhibitory activity. Our results should facilitate future experimental effects to find novel drugs for use against bovine brucellosis.

## 1. Introduction

*Brucella* spp. are important zoonotic pathogens worldwide [1,2,3]. Bovine brucellosis, which is primarily caused by *Brucella abortus*, represents a major problem for livestock industry development worldwide and is also a threat to human health in many developing and underdeveloped countries [4]. Gentamicin is the first-line antibiotic recommended for the treatment of brucellosis [5]. However, given the long duration of chemotherapy for brucellosis and the serious side effects associated with gentamicin [6], novel alternative agents with new protein targets are urgently required.

Aminoacyl-tRNA synthetases (aaRSs), which play key roles in translation, catalyze the covalent attachment of amino acids to their cognate tRNAs, yielding aminoacyl tRNAs (aa-tRNA or charged tRNA) for protein synthesis [7,8,9]. Because of their pivotal role in translation, aaRSs are considered to be among the most promising targets for antibiotic development in pathogenic species [10,11]. Mupirocin, which inhibits bacterial isoleucyl-tRNA synthetase (IleRS), was approved for the topical treatment of impetigo due to *Staphylococcus aureus* and *Streptococcus pyogenes* [12]. In addition to mupirocin, whole-cell screening of natural products has identified multiple aaRS inhibitors with antibacterial activity [12], including borrelidin (threonyl-tRNA synthetase, ThrRS) [13], granaticin (leucyl-tRNA synthetase, LeuRS), indolmycin (tryptophanyl-tRNA synthetase, TrpRS) [14], ochratoxin A (phenylalanyl-tRNA synthetase, PheRS), and cispentacin (prolyl-tRNA synthetase, ProRS) [15]. 

Virtual screening, a complementary approach to high-throughput screening (HTS) [16,17,18,19], facilitates discovery of novel and potential hits from large databases of diverse compounds by docking the compounds to the active site of a target protein [20,21,22,23,24,25]. This approach dramatically reduces the number of compounds that must be tested [26,27,28,29,30]. This technique has been successfully employed for the discovery of novel drugs [31,32,33,34,35,36].

This study was aimed at elucidating the 3D structural features of ThrRS from *Brucella abortus* (BaThrRS) and predicting interaction sites for substrates and inhibitors. To date, no experimentally determined 3D structures of *Brucella abortus* aaRSs have been published, and the rate at which aaRS structures are solved is insufficient to meet the need for development of drugs against brucellosis. Therefore, we used homology modeling to build a 3D structure of *Brucella abortus* aaRSs. Further refinement was achieved by subjecting the 3D model to molecular dynamics (MD) simulations. We also performed molecular docking studies to analyze the interactions among BaThrRS and its ligands, which should facilitate the design of novel drugs for the treatment of brucellosis. The 3D model of ThrRS obtained by comparative modeling analysis [37,38] provides insight into the influence of key amino acids on the enzyme’s activity and their interactions with ligands, and such models can help to design and forecast the ability of novel molecules to inhibit translation.

## 2. Results and Discussion

### 2.1. Sequence Alignments and Molecular Modeling

**Figure 1:**
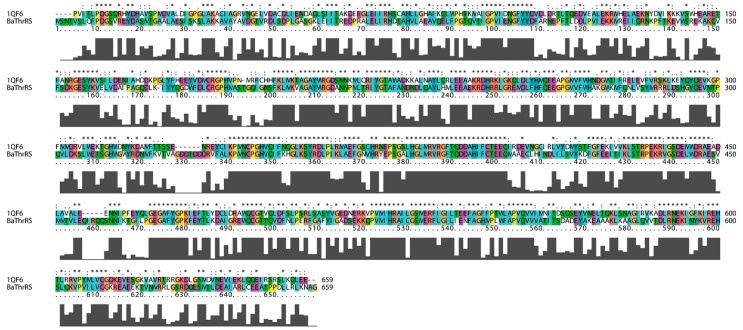
Sequence alignment of threonyl-tRNA synthetases from *Brucella abortus* (BaThrRS) and *Escherichia coli* (EThrRS) (sequence identity, 51%).

From the BLASTp matches of BaThrRS, we selected the structure of ThrRS from *Escherichia coli* (EThrRS) (PDB code 1QF6) [39] as the modeling template. Above 50% identity, models tend to be reliable, with only minor errors in side chain packing and rotameric state [40]; these two proteins share 51% sequence identity, sufficient to construct a reliable model. Sequence alignment was performed using Clustal X 2.0 [41] for homology modeling (Figure 1). The results revealed that the residues of the active site were conserved (EThrRS: Cys334, Arg363, Glu365, Met374, Arg375, Val376, Phe379, Gln381, His385, Gln479, Cys480, Thr482, His511, Gly516, Ser517, and Arg520; corresponding residues in BaThrRS: Cys343, Arg372, Glu374, Met383, Arg384, Val385, Phe388, Gln390, His394, Gln493, Cys494, Thr496, His525, Gly530, Ser531, and Arg534).

The coordinates of the crystal structure of EThrRS were used as a template to build the BaThrRS structure. The 3D model of BaThrRS was constructed with Modeller 9.16 [37,38]. To determine the optimal conformation of the BaThrRS model, further refinement was achieved by MD simulation for 20 ns. The final refined model was evaluated by stereochemical quality checking.

### 2.2. Validation of the Homology Model

**Figure 2:**
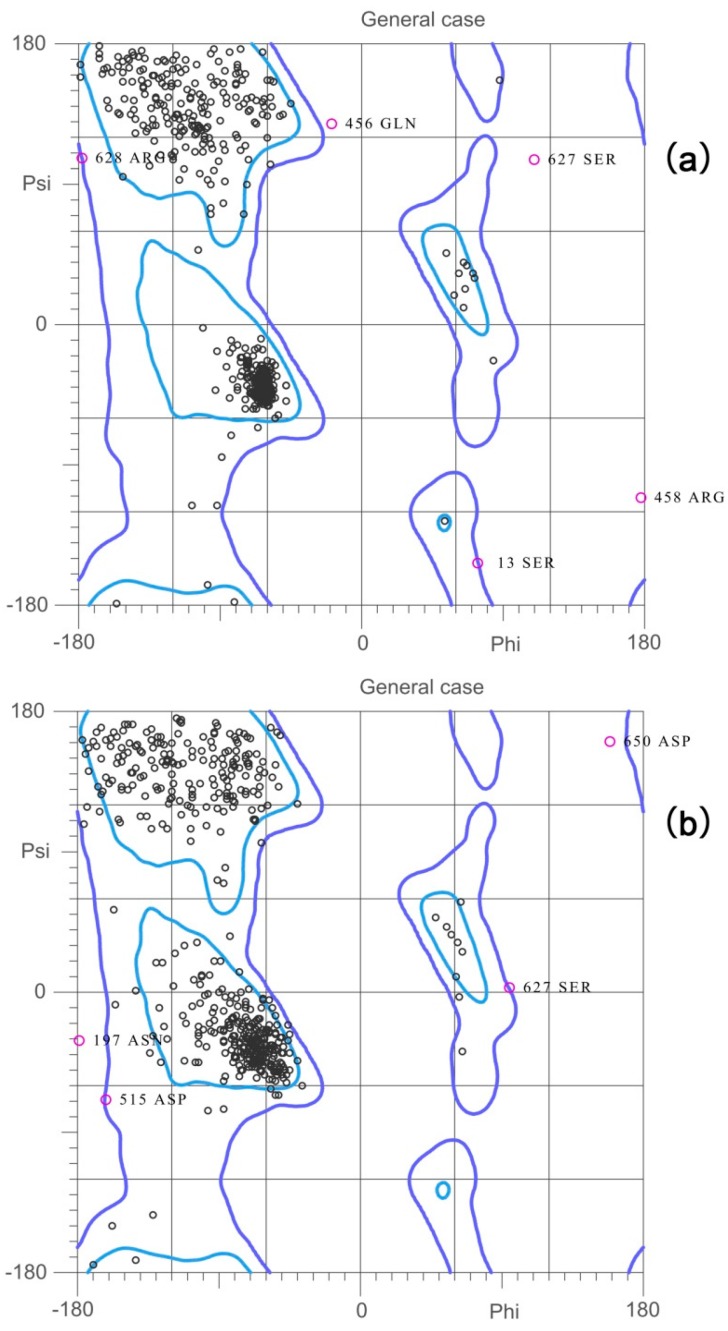
Ramachandran plots of (**a**) initial 3D structure and (**b**) final 3D structure of BaThrRS.

The first validation was carried out using Ramachandran plot calculations, computed with the MolProbity 4.3 software, which checks the detailed residue-by-residue stereochemical quality of a protein structure [42]. Then, overall quality factor for nonbonded interactions was checked by ERRAT [43]. Good high resolution structures generally produce ERRAT values around 95 or higher. For lower resolutions (2.5 to 3 Å) the average overall quality factor is around 91. Verify3D [44,45], which is a web-based tool that helps in the evaluation of a 3D model compared with its one-dimensional amino acid sequence, was also used. For a reliable model, the Verify3D value should be at least 80%. The results are shown in Figure 2 and Table 1. Before optimization, 95.1% (623/655) of all residues were in favored regions, 98.9% (648/655) were in allowed regions, and 1.07% were in disallowed regions. The ERRAT score was 76.425. Verify3D revealed that 90.27% of the residues had average 3D–1D scores. After refinement of the model, 92.2% (604/655) of all residues were in favored regions, 99.2% (650/655) were in allowed regions, and 0.76% were in disallowed regions. The ERRAT score was 85.440. Verify3D revealed that 93.76% of the residues had average 3D–1D scores. After optimization, the overall quality factors were increased and the error values were decreased by satisfying special constraints.

The template, 1QF6, had a Ramachandran plot similar to that of the final model: 98.7% of residues in allowed regions vs. 1.25% in disallowed regions. The ERRAT score for the template was 90.047. Verify3D revealed that 99.53% of the residues in 1QF6 had average 3D–1D scores. Thus, the geometric properties of the backbone conformation, residue interactions, residue contacts, and energy profile of the final model were similar to those of 1QF6, suggesting that the homology model of BaThrRS was reasonable and suitable for examination of protein-substrate and protein-inhibitor interactions, as well as for virtual screening.

### 2.3. Identification of Substrate-Binding Region in BaThrRS

ThrRS enzymes have a common substrate, ATP (ZINC18456332). The adenine moiety of ATP is sandwiched between Phe379 and Arg520 of EThrRS, and is specifically recognized by Ser517, which interacts with the N3 atom of ATP, and Glu365, which interacts with the N6 amino group. The ribose moiety is in the C3′-endo conformation, with its 2′ and 3′ hydroxyl groups essentially bound to Gln479 with two hydrogen bonds. The α-phosphate is stabilized by Arg363, which is also responsible for binding the carboxylate group of the cognate amino acid [39]. Therefore, the substrate-binding region in BaThrRS consists of Arg372, Glu374, Phe388, Gln493, Ser531, and Arg534, which correspond to residues Arg363, Glu365, Phe379, Gln479, Ser517, and Arg520 in EThrRS.

The cavity volume estimated by CASTp [46] is dependent on the radius of the probe sphere. A probe radius of 1.4 Å outlined substrate-binding cavities of 10,252 Å^3^ and 12,968 Å^3^ for BaThrRS and 1QF6, respectively. Thus, the active site of BaThrRS is smaller than that of EThrRS, which may indicate that the affinities of ligands with BaThrRS may be different from those of same ligands with EThrRS.

The residues of BaThrRS and EThrRS that participate in their respective ATP-binding sites are listed in Table 2. All of these residues are conserved, reflecting the high sequence identity (51%) between the two proteins. The center of mass of the pocket containing these residues, as determined by the docking study, was selected as the grid center (48.69 Å × 69.26 Å × 76.442 Å).

### 2.4. Docking Study

We docked the substrate ATP and the selective inhibitor borrelidin (ZINC30729522) to BaThrRS using AutoDock Vina [47]. The grid size for AutoDock Vina is 50 Å × 50 Å × 50 Å. The docking scores for ATP and borrelidin were −9.8 kcal/mol and −9.7 kcal/mol, respectively, suggesting that the binding of ATP is similar to that of borrelidin. 

**Figure 3:**
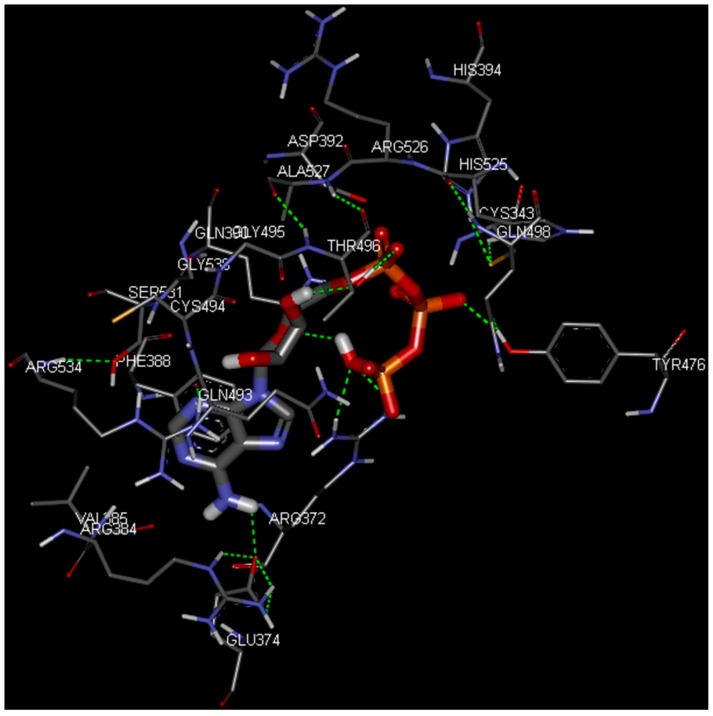
Predicted binding mode of ATP, as determined by docking of the ligands in the BaThrRS homology model.

**Figure 4:**
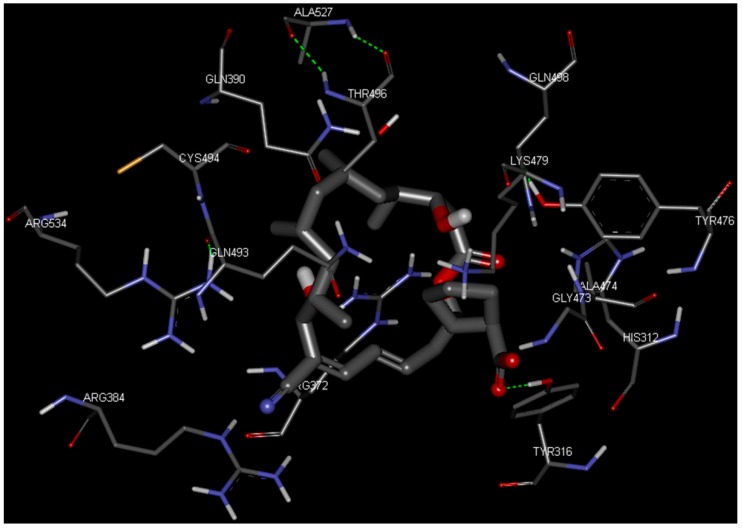
Predicted binding mode of borrelidin, as determined by docking of the ligands in the BaThrRS homology model.

**Figure 5:**
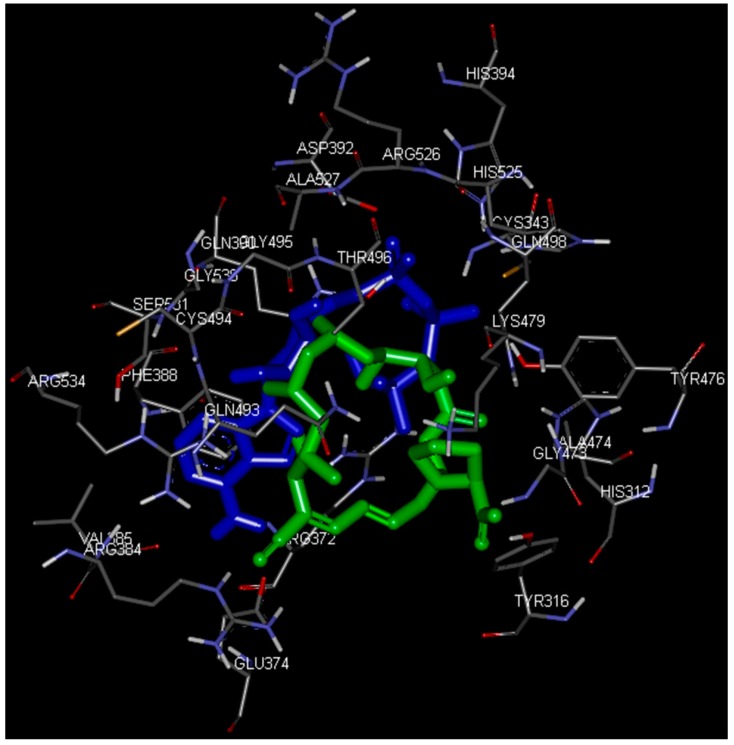
Predicted binding mode of ATP (blue) and borrelidin (green), as determined by docking of the ligands in the BaThrRS homology model.

Figure 3 and Figure 4 depict the docking of ATP and borrelidin, respectively, to BaThrRS. As shown in Figure 5, ATP (blue) is in the active site pocket, whereas borrelidin (green) is located near the ATP, also in accordance with a previous report [48]. 

Hydrogen bonds may play important roles in substrate binding. ATP and BaThrRS form eight hydrogen bonds, as seen in Figure 3. Arg372 forms three hydrogen bonds; Thr496 forms two; and Glu374, Tyr476, and Gln390 form one each. Phe388 engages in a π–π stacking interaction with ATP, and Arg534 forms a cation–π interaction. Therefore, Arg372, Thr496, Glu374, Tyr476, Gln390, Phe388, and Arg534 may be more important residues for ATP binding. The ATP is surrounded by Cys343, Arg372, Glu374, Arg384, Val385, Phe388, Gln390, Asp392, His394, Tyr476, Gln493, Cys494, Gly495, Thr496, Gln498, His525, Arg526, Ala527, Gly530, Ser531, and Arg534, most of which are consistent with predictions based on the sequence alignment of BaThrRS and EThrRS. These findings indicate that docking with AutoDock Vina may be used to predict the binding site and binding mode of ATP in BaThrRS. Hence, we used AutoDock Vina for further virtual screening of BaThrRS inhibitors. In selecting hits from the screen, we required that the scores of available novel inhibitors be lower than that of borrelidin (−9.7 kcal/mol).

### 2.5. High-Throughput Virtual Screening Procedure and Docking of the Best Inhibitor to BaThrRS

**Figure 6:**
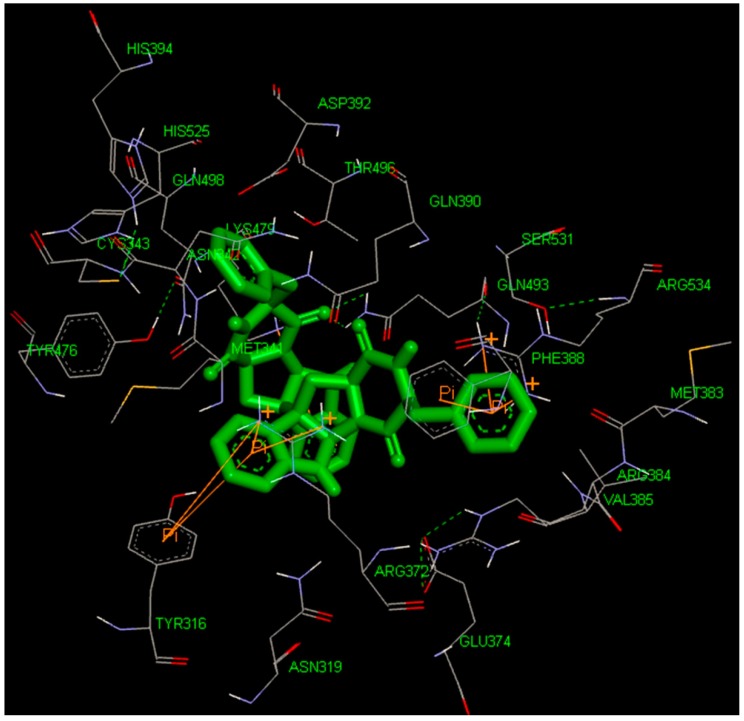
Predicted binding mode of ZINC27215482, as determined by docking of the ligands in the BaThrRS homology model.

To identify new and potent inhibitors against BaThrRS, we performed docking-based virtual screening on the active site of BaThrRS. The ZINC Natural Products Database (NPD) [49], which includes 11,247 compounds, was used for virtual screening using AutoDock Vina. The screen yielded 99 candidate compounds whose scores were not greater than −11 kcal/mol. The top hits, with scores lower than −12 kcal/mol, are listed in Table 3. It is clear from these results that the screened compounds have stronger interactions and better docking scores than the selective inhibitor borrelidin (−9.7 kcal/mol). We docked the best inhibitor, ZINC27215482 (−12.6 kcal/mol) to BaThrRS and further analyzed its interactions with key amino acid residues. Figure 6 shows the binding mode of ZINC27215482 in BaThrRS. Gln493 forms two hydrogen bonds with the inhibitor, Tyr316 and Phe388 engage in π–π interactions, and Arg534 forms two cation–π interactions. Because cation–π interactions can also contribute significantly to intermolecular contacts and interactions with ligands, it has been proposed that they should be considered alongside more conventional hydrogen bonds, salt bridges, and hydrophobic effects in any analysis of protein structure [50]. As discussed before, the cation–π interaction stabilizes the inhibitor–enzyme complex. As shown in Figure 6, ZINC27215482 was surrounded by Tyr316, Asn319, Met341, Asn342, Cys343, Arg372, Glu374, Met383, Arg384, Val385, Phe388, Gln390, Asp392, His394, Tyr476, Lys479, Gln493, Thr496, Gln498, His525, Ser531, and Arg534. These results serve as a guide for identifying the key residues around the best inhibitor and the interactions between BaThrRS and the best inhibitor, which could be used to develop novel drugs against bovine brucellosis.

## 3. Materials and Methods

### 3.1. Molecular Modeling

The 658-amino acid sequence of the target protein, BaThrRS, was obtained from the National Center for Biotechnology Information (NCBI) Protein Database (NCBI Reference Sequence: WP_002969106.1) [51]. The template protein for modeling was EThrRS (PDB ID 1QF6; sequence identity, 51%). The online search was conducted using the BLASTp algorithm [52,53]. Modeller 9.16 [37,38] was employed to build the 3D homology model. Molecular dynamics simulations were carried out using the Gromacs 5.1 software [54] with the AMBER-03 all-atom force field. Temperature was kept constant at 300 K. The protein was solvated using a box of simple point charge (SPC) water molecules extending at least 10 Å from the boundary of any protein atom. An integration step of 2 fs was used. Non-bonded interactions were calculated using a cutoff of 8 Å. Long-range electrostatic interactions were calculated by Particle–Mesh Ewald summation with grid spacing of 1.2 Å and cubic interpolation. After 1000 steps of steepest-descent energy minimization with protein heavy atoms frozen, the solvent and ions were equilibrated by 0.5 ns MD simulation, with protein heavy atoms subjected to harmonic constraints under a force constant of k = 1000 kcal·mol^−1^·nm^−2^. Finally, the production run was carried out for 20 ns, storing the coordinates of all atoms every 100 picoseconds for further analysis.

### 3.2. Assessment of the Homology Model

To obtain an accurate homology model, it is very important that appropriate quality assessment steps are built into the process. Therefore, in the modeling phase, model quality was assessed based on the geometric properties of the backbone conformation, residue interactions, residue contacts, and energy profile of the structure. Evaluation methods included ERRAT [43], Verify3D [44,45], and MolProbity [42]. ERRAT [43] was used for checking overall quality factor for nonbonded interactions. Verify3D [44,45] is a web-based tool that helps in the evaluation of a 3D model compared with its one-dimensional amino acid sequence. MolProbity software was used for Ramachandran plot calculations, which checks the detailed residue-by-residue stereochemical quality of a protein structure [42].

### 3.3. Binding Pocket Analyses

The volume of the binding pocket was computed using the CASTp server [46] with default settings. The probe radius was set as 1.4 Å, and moreover, HET groups in the calculation were excluded in the default settings.

### 3.4. Validation of the Model by Docking Analysis

A docking study was conducted to evaluate the predictive ability of the BaThrRS homology model and its suitability for use in structure-based drug design studies. The structures of borrelidin (ZINC30729522) and ATP (ZINC18456332), a substrate of ThrRS enzymes, were obtained from the ZINC database [49]. Ligands and receptor molecules were prepared using AutoDock Tools 1.5.6. [55] Since the amino acids that comprise the ATP-binding site of EThrRS are known, the amino acids that comprise the ATP-binding site of BaThrRS were predicted by comparing the amino acid sequence of its active site with that of EThrRS. A grid map including all amino acids within the ATP-binding site of BaThrRS was defined as follows: size, 50 Å × 50 Å × 50 Å; grid center, 48.69 Å × 69.26 Å × 76.442 Å; spacing between grid points, 0.375 Å. AutoDock Vina [47] was used to perform docking.

### 3.5. Virtual Screening

The ZINC NPD, [49] which includes 11,247 compounds, was employed for virtual screening using the 3D structure of BaThrRS. All compounds in the database were processed (removal of all inorganic counterions, addition of hydrogen atoms, deprotonation of strong acids, protonation of strong bases, and generation of stereoisomers), and then subjected to the same docking procedure. After docking, compounds with docking scores lower than −11 kcal/mol were chosen for further analysis.

## 4. Conclusions

This paper describes how a reliable and reasonable 3D structure of BaThrRS was built using homology modeling techniques and MD methods. To determine the residues important for substrate (ATP) binding, we docked ATP to the protein. Arg372, Thr496, Glu374, Tyr476, Gln390, Phe388, and Arg534 were found to be important residues for ATP binding. Virtual screening of the ZINC NPD yielded 99 novel compounds. The scores of the seven top-scoring docking hits were lower than −12 kcal/mol (lower than that of ATP and borrelidin), indicating that they represent potential inhibitors of BaThrRS. We hope that our results will aid in future efforts to design inhibitors of BaThrRS for use in the treatment of brucellosis.

## Figures and Tables

**Table 1:** Validation of various 3D structures.

Validation	BaThrRS (0 ns)	BaThrRS (20 ns)	1QF6
Favored regions	95.1% (623/655)	92.2% (604/655)	87.8% (561/639)
Allowed regions	98.9% (648/655)	99.2% (650/655)	98.7% (631/639)
Ramachandran outliers	1.07% (7/655)	0.76% (5/655)	1.25% (8/639)
Verify3D	90.27%	93.76%	99.53%
ERRAT	76.425	85.440	90.047

**Table 2:** Conserved binding-site residues of 1QF6 and BaThrRS.

1QF6	BaThrRS
Cys334	Cys343
Arg363	Arg372
Glu365	Glu374
Met374	Met383
Arg375	Arg384
Val376	Val385
Phe379	Phe388
Gln381	Gln390
His385	His394
Gln479	Gln493
Cys480	Cys494
Thr482	Thr496
His511	His525
Gly516	Gly530
Ser517	Ser531
Arg520	Arg534

**Table 3:** Free energy of binding between BaThrRS and novel inhibitors.

ZINC ID	Score (kcal/mol)	Structure
ZINC27215482	−12.6	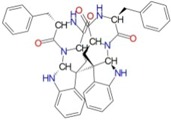
ZINC67910544	−12.4	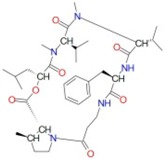
ZINC42805205	−12.4	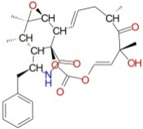
ZINC72320615	−12.3	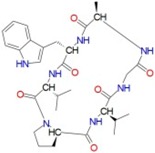
ZINC72320626	−12.2	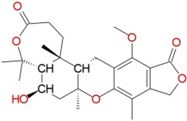
ZINC35270978	−12.1	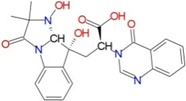
ZINC35458951	−12.1	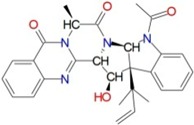

## Abbreviations

ThrRS	threonyl-tRNA synthetase
BaThrRS	*Brucella abortus* threonyl-tRNA synthetase
PDB	Protein Data Bank
ATP	adenosine triphosphate
tRNA	transfer RNA
aaRS	aminoacyl-tRNA synthetase
EThrRS	*Escherichia coli* threonyl-tRNA synthetase
NPD	Natural Products Database
